# Whole Genome Sequencing of Field Isolates Reveals a Common Duplication of the Duffy Binding Protein Gene in Malagasy *Plasmodium vivax* Strains

**DOI:** 10.1371/journal.pntd.0002489

**Published:** 2013-11-21

**Authors:** Didier Menard, Ernest R. Chan, Christophe Benedet, Arsène Ratsimbasoa, Saorin Kim, Pheaktra Chim, Catherine Do, Benoit Witkowski, Remy Durand, Marc Thellier, Carlo Severini, Eric Legrand, Lise Musset, Bakri Y. M. Nour, Odile Mercereau-Puijalon, David Serre, Peter A. Zimmerman

**Affiliations:** 1 Unité d'Epidémiologie Moléculaire du Paludisme, Institut Pasteur du Cambodge, Phnom Penh, Cambodia; 2 Genomic Medicine Institute, Cleveland Clinic Lerner Research Institute, Cleveland, Ohio, United States of America; 3 Direction de la lutte contre les maladies infectieuses, Ministère de la santé, du planning familial et de la protection sociale du Madagascar, Antananarivo, Madagascar; 4 Laboratoire de Parasitologie-Mycologie, Hôpital Avicenne, AP-HP, Bobigny, France; 5 National Center for Malaria Research, AP-HP, CHU Pitie Salpêtrière, Paris, France; 6 Department of Infectious, Parasitic and Immunomediated Diseases, Istituto Superiore di Sanità (ISS), Rome, Italy; 7 Laboratoire de parasitologie, National Reference Centre of Malaria Resistance in French Guiana and West Indies, Institut Pasteur de la Guyane, Cayenne, French Guiana; 8 Blue Nile National Institute for Communicable Diseases, University of Gezira, Wad Medani, Sudan; 9 Unité d'lmmunologie Moléculaire des Parasites, Institut Pasteur, Paris, France; 10 Center for Global Health and Diseases, Case Western Reserve University, Cleveland, Ohio, United States of America; New York University, United States of America

## Abstract

**Background:**

*Plasmodium vivax* is the most prevalent human malaria parasite, causing serious public health problems in malaria-endemic countries. Until recently the Duffy-negative blood group phenotype was considered to confer resistance to vivax malaria for most African ethnicities. We and others have reported that *P. vivax* strains in African countries from Madagascar to Mauritania display capacity to cause clinical vivax malaria in Duffy-negative people. New insights must now explain Duffy-independent *P. vivax* invasion of human erythrocytes.

**Methods/Principal Findings:**

Through recent whole genome sequencing we obtained ≥70× coverage of the *P. vivax* genome from five field-isolates, resulting in ≥93% of the Sal I reference sequenced at coverage greater than 20×. Combined with sequences from one additional Malagasy field isolate and from five monkey-adapted strains, we describe here identification of DNA sequence rearrangements in the *P. vivax* genome, including discovery of a duplication of the *P. vivax* Duffy binding protein (PvDBP) gene. A survey of Malagasy patients infected with *P. vivax* showed that the PvDBP duplication was present in numerous locations in Madagascar and found in over 50% of infected patients evaluated. Extended geographic surveys showed that the PvDBP duplication was detected frequently in vivax patients living in East Africa and in some residents of non-African *P. vivax*-endemic countries. Additionally, the PvDBP duplication was observed in travelers seeking treatment of *vivax* malaria upon returning home. PvDBP duplication prevalence was highest in west-central Madagascar sites where the highest frequencies of *P. vivax*-infected, Duffy-negative people were reported.

**Conclusions/Significance:**

The highly conserved nature of the sequence involved in the PvDBP duplication suggests that it has occurred in a recent evolutionary time frame. These data suggest that PvDBP, a merozoite surface protein involved in red cell adhesion is rapidly evolving, possibly in response to constraints imposed by erythrocyte Duffy negativity in some human populations.

## Author Summary

Malaria results from infection of human red blood cells (RBC) by *Plasmodium* parasite's merozoite. For *Plasmodium vivax* the process of RBC invasion has been hypothesized to depend on interactions between the parasite's Duffy binding protein (PvDBP) and human Duffy blood group antigen because Duffy-negative people (most often people of African descent) were shown to be highly resistant to RBC infection and disease. Over the past five years, researchers are reporting with increasing frequency that Duffy-negative individuals are infected with *P. vivax*. This raises new questions as to how *P. vivax* infects the RBC when the Duffy blood group antigen is not available. Here we show that the parasite's Duffy binding protein gene has been duplicated in multiple *P. vivax* strains, especially at high prevalence in Madagascar. The specificity and prevalence of this polymorphism suggest that the parasite genome has responded to the barrier of Duffy negativity through the duplication of the PvDBP gene. Our results indicate that the PvDBP duplication is a recent event and provide novel research avenues to understand alternative pathways for *P. vivax* RBC invasion.

## Introduction

Merozoite contact and tight junction formation with the red blood cell (RBC) initiate the cycle of events that result in human malaria [1]. Evidence that these events require specific molecular interactions was revealed through *in vitro* and *in vivo* studies demonstrating that *P. knowlesi* and the highly prevalent, human parasite, *P. vivax* were unable to cause infection of RBCs that did not express the Duffy blood group antigen [2], [3]. With these observations and advent of molecular biological methods, the first of the so-called malaria parasite invasion ligands identified was the *P. knowlesi* Duffy binding protein that is expressed in the micronemes located at the merozoite apex [4]. This parasite protein has been implicated in interactions leading to formation of a tight junction between parasite and RBC membranes, through which the parasite propels itself, resulting in invagination of the parasitophorous vacuole, where the parasite will reside and develop. The events governed by DBP-Duffy antigen interaction were shown to fail when *P. knowlesi* merozoites were allowed to interact with Duffy-negative human RBCs [5], and subsequently when *P. knowlesi* DBP knockout mutants were allowed to interact with Duffy-positive human RBCs [6]. These observations have contributed to the paradigm that Duffy negativity confers resistance to *P. vivax* blood stage infection and vivax malaria [7].

Vivax malaria is considered to be rarely transmitted or absent from Sub-Saharan Africa where most individuals are Duffy blood group-negative. We have therefore been surprised by our recent findings in Madagascar showing that *P. vivax* was commonly able to infect Duffy-negative human red blood cells [8]. These results have confirmed PCR-based studies that have reported *P. vivax* positivity in Duffy-negative people in South America [9], and from East [10], Central [11] and West African [12] locations. In order for *P. vivax* to infect Duffy-negative RBC, it is necessary to consider how the invasion events occur without interaction between *P. vivax* Duffy binding protein (PvDBP) and the Duffy blood group protein. Given the observed molecular specificity of the parasite-host interaction, our findings have suggested that it may be necessary to identify a new parasite invasion ligand, or determine if PvDBP polymorphisms enable this protein to interact with different invasion receptors on the human RBC surface.

Multiple challenges limit the functional studies needed to identify and characterize components of an alternative pathway enabling *P. vivax* to invade Duffy-negative reticulocytes. In view of difficulties of *P. vivax in vitro* culture, we have chosen to investigate genetic polymorphism among *P. vivax* strains through whole genome sequencing of field isolates to compare variation from Madagascar where Duffy-negative people are known to experience vivax malaria and from Cambodia where Duffy-negativity has not been observed [13]. The first of these studies characterized SNPs throughout the *P. vivax* genome, revealed approaches for identifying multiple strains within individual infections and illustrated extensive allele sharing globally, presumably through distribution of dormant liver-stage hypnozoites [14]. Here, using strategies to identify sequence rearrangements in Illumina short-read sequence when compared to the Sal I reference sequence [15], we seek to determine if any gene rearrangements uniquely characterize P. vivax from Madagascar.

## Materials and Methods

### Samples and ethics statement

All patient samples were anonymized and obtained as part of on-going studies in accordance with human studies protocols IRB N°035-CE/MINSAN (Comité d'Ethique du Ministère de la Santé de Madagascar, June 30th 2010) and IRB N°160 NECHR (National Ethics Committee for Health Research – Cambodia, October 28th 2010). We collected blood samples from one patient from Madagascar (M15) in 2010. Clinical isolates of *P. vivax* were collected from symptomatic malaria-infected patients: from 1997–2009 for travelers returning to France from various countries (National Reference Center for Malaria, Paris, France), in 2005–2008 (Madagascar), in 2010 (Cambodia), in 2000–2003 (French Guiana), in 2007 (Sudan) and in 2000–2003 (Middle East countries).

Institutional review boards or equivalent committee(s) of University Hospitals of Cleveland, Pasteur Institute and Madagascar Ministry of Health approved the study. All study participants provided informed consent and when participants were children parents/guardians provided consent; informed consent given was written.

### Library preparation and genome sequencing

We sequenced DNA extracted from the M15 sample using the same protocol as described earlier [14]. Briefly, we first sheared DNA extracted from the M15 sample into 250–300 bp fragments using a Covaris S2 sonicator. We then repaired the ends of the fragmented molecules and added a 3′ terminal adenine overhang to enable ligation of Illumina paired-end adapters. Finally, we selected ligated fragments of ∼300 bp using an E-gel (Invitrogen) and amplified the library by 12 cycles of PCR using Phusion Taq. We verified the quality and quantity of the libraries using an Agilent Bioanalyzer and qPCR using Illumina primers. We sequenced this library on one lane of an Illumina HiSeq 2000 to generate 76 million paired-end reads of 100 bp.

To take into account recent developments in alignment software [16], we mapped all reads from *P. vivax* samples sequenced by us [14] and others [17] to the human (UCSC build hg18, [18]) and the *P. vivax* Sal I strain [15] reference genome sequences using bowtie 2. We did not include in our analyses an isolate from Peru previously sequenced [19] due to its lower coverage. For each of the monkey-adapted strains sequenced at the Broad Institute, we used one lane of sequence data retrieved from NCBI Short Read Archive: SRR332569 (Brazil), SRR332913 (India VII), SRR332413 (Mauritania I) and SRR332562 (North Korea). We mapped separately the ends of each read pair (i.e., without treating them as paired-end reads) allowing for zero mismatch in the 15 nucleotide long seed. We allowed for up to 20 consecutive seed extensions and up to 3 re-seedings. We only considered in our analyses read pairs for which each end mapped uniquely in the *P. vivax* genome (i.e., a unique best location on the final assembly of the 14 chromosomes). We identified read pairs that mapped to the exact same positions (and could represent molecules amplified during the library preparation) and randomly discarded all but one pair using custom Perl scripts to parse the alignment output from bowtie 2 (all scripts are available upon request).

### Identification of SNPs and DNA sequence rearrangements

To identify SNPs, we followed the procedure described in Chan et al [14].

To identify DNA sequence rearrangements across the genome we relied solely on paired-end read configuration (i.e. not sequence coverage) and screened for paired-end reads that did not map in the expected configuration (i.e. head-to-head within 1 kb from each other) using custom Perl scripts to parse the alignment output. We classified these paired-end reads in the following categories: i) reads mapping head-to-head but distant by more than 1 kb, ii) reads mapping in a head-to-tail configuration and iii) reads mapping tail-to-tail (**Figure S1**). Note that the distance of 1 kb was selected to identify putative deletions and chosen to focus on relatively large DNA sequence deletions and to minimize false positives caused by DNA fragments slightly greater than 300 bp that had not been excluded during size selection. We also excluded from our analyses 5 kb at the telomeric end of each chromosome to concentrate on non-repetitive DNA sequences. For each category of rearrangement (i.e., deletion, inversion and duplication), we calculated the number of pairs in the indicative configuration in each non-overlapping 100 bp window in the genome. To determine the significance cut-off for each type of rearrangement, we assumed that windows with 0 or 1 read (respectively, U0 and U1) were mainly background and fitted a Poisson distribution to represent the distribution of reads per windows expected by chance (with λ = U1/U0). We then compared the observed number of 100 bp windows with a given number of reads to the expected number of windows with the same number of reads under the null model and calculated a false discovery rate (FDR). Windows with FDR<1% were considered significant and further analyzed. To avoid analyzing library artifacts, we focused here on rearrangements greater than 1,000 bp and smaller than 100 kb.

### PvDBP duplication-specific PCR assay

We designed two primer pairs to amplify the region containing the estimated breakpoints of the duplicated region based on the unusual paired-end mapping. Primer pair 5′-CCATAAAAGGTAGGAAATTGGAAA-3′ (AF) and 5′-GCATTTTATGAAAACGGTGCT-3′ (AR) are designed to amplify a 613 bp region surrounding the breakpoint at position 382,640 and primer pair 5′-TCATCGAGCATGTTCCTTTG-3′ (BF) and 5′-TTGCACGTACTCGAAACTCAG-3′ (BR) amplifies a 643 bp region surrounding the breakpoint at position 390,817. These two primer pairs amplify these regions in samples with and without the duplication and were used as positive controls. In samples with the duplication, BF and AR primers amplified the junction between the original and duplicated region yielding a 584 bp product. In samples without duplication, the primers are positioned in opposite directions and will not amplify any DNA. This product was subsequently sequenced using Sanger sequencing chemistry and the exact duplication breakpoints were identified. PCR was performed using Phusion Taq with the following conditions: 95°C for 3 min, followed by 35 cycles of 95°C for 30 s, 56°C for 30 s, and 72°C for 30 s.

## Results and Discussion

### Whole genome sequencing and sequence rearrangements

We have recently published the complete genome sequence from five field isolates, two from Madagascar (M08 & M19) and three from Cambodia (C08, C127 & C15) [14]. Genome sequencing, at very high sequence coverage (>70×) allowed identification of >80,000 SNPs throughout the genome. We have now sequenced, at similar coverage, one additional field isolate from Madagascar (later referred to as M15), and used sequence information from all these samples and from monkey-adapted strains [17] (**Table S1**) to investigate a second type of DNA polymorphism: variations in DNA sequence copy number and organization [20], [21] that can underlie important functional differences [22].

**Figure 1:**
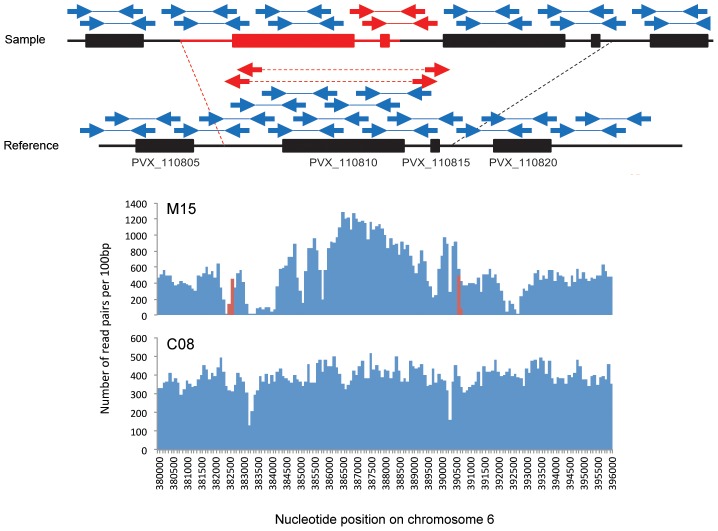
Duplication of the Duffy binding protein gene. The top panel schematically shows the duplicated region and resulting read mapping on the Sal I reference genome. The bottom panels show, for M15 (Madagascar) and C08 (Cambodia), the number of read pairs per 100 bp non-overlapping bins (y-axis) that mapped head-to-head within the expected distance from each other (in blue) and read pairs that mapped tail-to-tail (in red). The x-axis corresponds to chromosome 6 between nucleotide positions 380,000 and 395,000. Note the elevated read coverage and presence of tail-to-tail read pairs in the M15 samples that are indicative of the PvDBP gene duplication and that are absent from the C08 sample (not duplicated).

Sequence rearrangements can be studied by analyzing variations in read coverage: duplicated sequences have increased coverage, while deleted regions in haploid genomes are indicated by a lack of coverage. Both types of sequence variation were observed in our data analysis, mostly localized to the telomeric regions of *P. vivax* chromosomes (see e.g. **Figure S2**). However, many factors that influence sequencing (e.g., GC content) can introduce biases in coverage that complicate genome-wide identification of sequence rearrangements. In addition, analyses based solely on sequence coverage are further complicated in patient samples by the presence of multiple parasite strains [14]: if a locus is deleted in a strain that accounts for 10% of the parasites in the blood, the sequence coverage would only decrease by 10% and be difficult to identify. We therefore focused instead on read pair arrangements that are indicative of sequence duplication, deletion, insertion or inversion (see **Figure S1** for details). Since these unusual arrangements occur very rarely by chance (see Materials and Methods), observations of multiple independent events in a region allowed us to reliably identify sequence rearrangements even if they occur in a minor strain (the power to detect a rare rearranged strain increasing with increased genome coverage). Across all samples (n = 12), we identified 19 putative deletions, 12 duplications and 7 inversions (**Table S2**). Most of these detected rearrangements occurred in multigene family clusters and may represent artifacts due to mismapping of the short reads in highly homologous regions. We discarded these regions from the current analysis and ended with six putative rearrangements involving non-repeated DNA sequences: five deletions and one duplication (**Table 1**). For example, a 2 kb deletion on chromosome 6 containing a Phist protein gene (*Pf-fam-b*, PVX_001700) was detected in the Belem strain as well as on a minor strain of C15 (**Figure S3**). The deletion at the telomeric end of chromosome 14 (**Figure S4**) was detected in multiple samples from various geographical areas. This deletion affected 7 kb of DNA containing the reticulocyte binding protein 2 like gene (PVX_101590). We have also identified a single tandem duplication encompassing the entire Duffy binding protein gene (PVX_110810) on chromosome 6 (**Figure 1**).

**Table 1:** Summary of all DNA sequence rearrangements identified.

Chromo-some	Start^a^	End^b^	Length^c^	Type^d^	Sal I	Belem	C08	C127	C15	M08	M15	M19	Brazil	India VII	Mauritania I	North Korea	Annotation
vivax02	14,100	18,100	4,000	Δ	-	-	-	-	-	-	-	-	-	-	-	5	pseudogene
vivax06	71,800	74,000	2,200	Δ	-	116	-	-	4	-	-	-	-	-	-	-	Phist protein
vivax09	1,536,000	1,537,600	1,600	Δ	-	-	-	-	-	-	-	-	-	-	-	82	none^e^
vivax14	780,100	781,500	1,400	Δ	-	-	-	-	-	-	-	-	-	-	-	20	none
vivax14	3,061,600	3,069,300	7,700	Δ	-	-	7	81	-	52	-	62	15	27	18	-	RBP2L
vivax06	382,600	390,700	8,100	2x	-	-	-	-	-	-	21	2	-	-	3	-	Duffy binding receptor

The table shows for each sample the number of read pairs in unusual configuration indicating putative DNA sequence rearrangements. Dashes (-) indicate that no read pairs supported a sequence rearrangement at this location. See also Table S2.

a Upstream chromosomal coordinate of the sequence rearrangement based on the Sal I reference sequence.

b Downstream chromosomal coordinate of the sequence rearrangement based on the Sal I reference sequence.

c Nucleotide length of the sequence rearrangement.

d Δ = deletion; 2x = duplication.

e none = no annotated gene.

### Identification of a tandem duplication of the Duffy binding protein gene in Malagasy *P. vivax* strains

**Figure 2:**
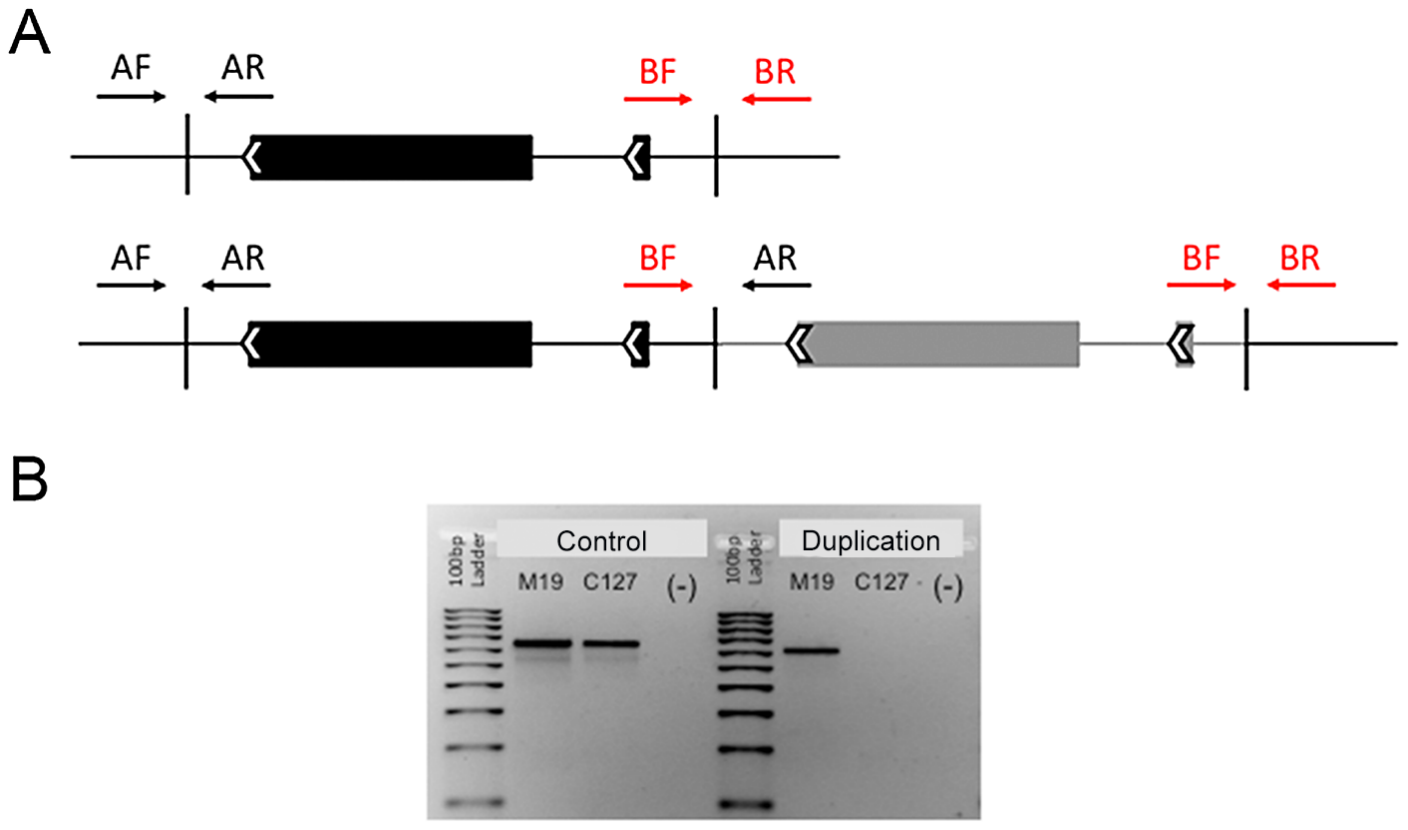
PvDBP gene duplication PCR diagnostic assay. **Panel A**: Diagram of the PvDBP duplication and orientation of PCR primers to amplify control upstream (AF+AR; F = forward, R = reverse) and control downstream (BF+BR) fragments. Only when the sequence is duplicated will BF+AR primers amplify a PCR product. **Panel B**: Agarose gel following PCR amplification using AF+AR (Control) and BF+AR (Duplication) primers. Control products were obtained from the Madagascar (M19) and Cambodia (C127) samples; (-) indicates no DNA amplification from negative control (water). Duplication products were observed in Malagasy but not in Cambodian samples analyzed by whole genome sequencing.

Unusual arrangements of the paired-ends of multiple reads revealed a duplication of the PvDBP gene in two of the three Malagasy samples (**Figure 1**): the large proportion of affected reads and the almost 2-fold increase in coverage in this region for the M15 sample indicated that the PvDBP duplication was present in the major *P. vivax* strain, while we predict that this duplication was carried by one of the minor clones of the M19 sample (**Figure S5**). In addition, similar read pair organization was detected (in the same location) in a minority of the Mauritania I sequences in sequence reported previously by Neafsey et al [17]. This observation suggests that this monkey-adapted strain may contain more than a single strain, including at least one strain carrying the PvDBP duplication, but this hypothesis will need to be tested in future analyses. The identification of this duplicated sequence was verified independently by a PCR assay designed to amplify specifically the sequence present at the junction of the tandemly duplicated gene sequences (**Figure 2**). This PCR assay confirmed our observation based on read pair arrangements that the two PvDBP copies were located next to each other and in the same orientation. The duplicated region included the entire PvDBP protein coding sequence, 2,858 bp downstream of the gene and 1,558 bp upstream of the translation start site, including a single-exon hypothetical protein (PVX_110815) and likely, the proximal promoter of the PvDBP gene. Overall, the duplicated region represented a total length of 8,177 bp. Using conventional Sanger sequencing of PCR products amplified using primers located on either side of the duplication (see **Figure 2B**), we were able to determine that the exact boundaries of this duplication coincided with long homopolymer stretches on either side (18 to 22 T residues) of the sequence rearrangement. Note that after cloning and sequencing the “junction” PCR products, we obtained a single DNA sequence (i.e., identical in all clones) suggesting that only two copies of the PvDBP genes (next to each other) were present in these samples. This is consistent with the doubling in sequence coverage we observed in the M15 sample where the duplication was carried by the dominant strain of the *P. vivax* infection.

To determine whether the two copies shared identical DNA sequence, we concentrated on DNA polymorphisms present in the M15 sample. Since the duplication was present in the dominant *P. vivax* clone of this sample (80% of this *P. vivax* infection; **Figure S6**), we expected that any position differing between the two copies would display an allele frequency of ∼40%. Fourteen positions (13 non-synonymous and 1 synonymous) differed between M15 and the Sal I PvDBP sequence (**Table 2**), 13 of these differences were supported by less than 10% of the reads (note that this is also true for P1052P where the allele frequency of 90% simply indicates that the reference allele is the most common allele among *P. vivax* parasite sequences present in the M15 sample). These low frequency polymorphisms likely represent differences between the major strain and some of the other *P. vivax* parasites present in M15. The last substitution (L17S) occurred at a frequency of 43%, suggesting that this position differed between the two tandemly repeated copies. Note that, across the entire genome, less than 0.005% of nucleotides (926 positions out of 19,981,509 positions analyzed) showed such intermediary allele frequencies in this sample. This substitution was only observed in one of the other samples sequenced, M19, where a minor strain carried the duplication. In the M19 sample, the L17S substitution was observed in 9 out of 206 reads (4%), an allele frequency consistent with this position being different between the two copies. Interestingly, all 14 nucleotide substitutions differentiating M15 and the Sal I PvDBP sequence were also observed in the M19 sample and one of the M19 PvDBP haploid sequences generated by cloning and Sanger sequencing was similar to the inferred M15 sequence for this locus (**Figure S7**). Overall, these findings suggested that the duplication of the PvDBP gene in these samples carries the same PvDBP haplotype and therefore resulted from a single and recent ancestral event.

**Table 2:** Single Nucleotide Polymorphisms in the PvDBP gene of the M15 sample.

Nucleotide Position	389210	388688	388604	388601	388109	388091	387951	387752	387399	387300	386389	386352	386190	385646
**Amino Acid change**	L17S	A146E	T174I	G175E	D339G	R345H	W392R	I458K	Q576K	K609E	K912N	D925N	Y979H	P1052P
**Ref** a **/Alt allele**	A/G	G/T	G/A	C/T	T/C	C/T	A/G	A/T	G/T	T/C	T/A	C/T	A/G	A/G
**Reads**	104/77	2/169	9/122	1/127	1/178	12/167	8/212	5/169	3/172	2/185	15/158	12/146	15/151	170/18
**Ref Allele Freq**	57	1	7	1	1	7	4	3	2	1	9	8	9	90

The table shows each polymorphic position in the PvDBP gene (PVX_110810), its resulting amino acid and reads supporting each allele at each position. Position 389210 is the nucleotide/amino acid change in the signal peptide likely differentiating the two PvDBP copies.

a The reference allele is the PVX_110810 sequence.

### The PvDBP duplication in Malagasy and non-Malagasy *P. vivax* infections

**Figure 3:**
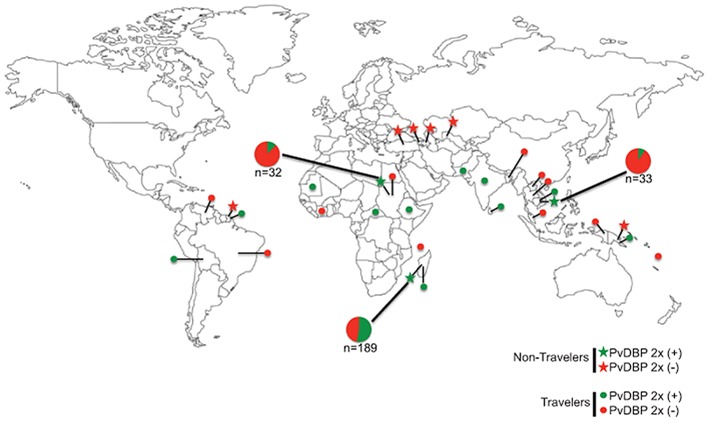
Global distribution of the PvDBP duplication allele in non-traveler (NT) and traveler (T) sample sets. Blood samples from NT (filled stars) and T (filled circles) show the locations where the PvDBP duplication was detected (green) and not detected (red). Three locations included samples from NT and individuals that were infected with *P. vivax* strains that carried the PvDBP duplication (Sudan, Madagascar and Cambodia). Details regarding sample sizes for all NT and T sample sets are provided in Table S1.

Given the importance of the PvDBP and Duffy blood group protein interaction during RBC invasion we evaluated the prevalence of the PvDBP duplication in *P. vivax* infections. For this survey we performed the duplication-specific PCR assay demonstrated in **Figure 2** on *P. vivax*-infected blood obtained from participants in field-based malaria epidemiology studies (non-travelers [NT], n = 389) and patients seeking treatment for malaria symptoms following recent travel to malaria-endemic countries (travelers [T], n = 110) (**Figure 3** and **Table S3**). Among NT, 189 *P. vivax*-infected people were from Madagascar and the PvDBP duplication was observed in 100 infections (52.9%). The PvDBP duplication was also detected in NT *P. vivax* infections from Sudan (4/32, 12.5%) and Cambodia (3/33, 9.1%), but not in Papua New Guinea (N = 83), in French Guiana (N = 10) or Middle Eastern countries (N = 42, Armenia, Turkey, Azerbaijan and Uzbekistan). The PvDBP duplication was also detected in *P. vivax*-infected blood samples from travelers returning from South America, Africa (includes Madagascar), Asia and Melanesia (**Figure 3** and **Table S3**).

These observations show that the highest prevalence of the PvDBP duplication was detected in Madagascar in areas where we previously observed the highest prevalence of Duffy-negative people infected with *P. vivax* [8]. Our results also show that *P. vivax* strains carrying the PvDBP duplication are present outside of Madagascar. This is consistent with our previous observations of a significant genome-wide allele sharing among South American, African and Asian regions [14]. Given recent global mapping of *P. vivax* endemicity that has integrated Duffy blood group polymorphism [13], [23], it is interesting that many of the locations where the PvDBP duplication has been detected are areas where Duffy-negativity is estimated to range from 45% (Ethiopia) to 95% (Mauritania).

### Associations between PvDBP duplication and Duffy genotype

Earlier studies have reported differences in susceptibility to *P. vivax* infection among Duffy-positive individuals. Lower susceptibility to *P. vivax* infection and vivax malaria has been associated with heterozygous Duffy-negative vs. homozygous Duffy-positive phenotypes [9], [24]–[26] and the Fy(a+b−) vs. Fy(a−b+) phenotypes [27], [28]. Our preliminary assessment of single-copy and duplicated PvDBP alleles based on *P. vivax* infections of Duffy-positive Malagasy non-travelers showed the following results. Among those infected with *P. vivax*, 45 were homozygous carriers of Duffy-positive alleles (FY*A/*A, 16; FY*A/*B, 19; FY*B/*B, 10) and 144 were heterozygous carriers of the FY*B^ES^ allele (FY*A/*B^ES^, 68; FY*B/*B^ES^, 76). The prevalence of the PvDBP duplication was higher in homozygous Duffy-positive (64.4%) vs. heterozygous Duffy-negative (48.6%) people, however this difference was not statistically significant (chi-square, 1df, p = 0.063). We observed no significant difference in distribution of the PvDBP duplication based on Duffy genotypes (FY*A/*A = 62.5% vs. FY*B/*B = 60.0%, chi-square, 1df, p = 0.899; FY*A/*B^ES^ = 45.7% vs. FY*B/*B^ES^ = 49.9%, chi-square, 1df, p = 0.499). These results show that *P. vivax* strains carrying the PvDBP duplication did not exhibit any significant predisposition in infection based on predicted Duffy-positive phenotypes. It will be important to monitor these relationships in future epidemiological studies designed to test these associations rigorously.

### Limitations of this study

It would be interesting to obtain the haplotypes of all strains carrying the PvDBP duplication to confirm that the duplication occurred only once in the genealogy of *P. vivax* and to date this event. Unfortunately, to date we have only been able to determine the haplotype carrying the duplication for the samples analyzed by whole genome sequencing (e.g., none of the 389 NT or 110 T samples have received this level of sequence analysis). Since many genetically different strains are present in most (if not all) patient samples, it is difficult to know which haplotypes correspond to the duplicated strain(s). We attempted to selectively amplify the duplicated haplotypes using a primer sitting on the duplication boundary but the resulting ∼600 bp sequence was identical in all isolates evaluated (and identical to the Sal I sequence). Prior amplification of the entire ∼15 kb duplicated region would enable such analyses but would require better quality DNA samples than was available. Alternatively, sequence capture (e.g., [29]) may provide an efficient method to select duplicated DNAs before sequencing.

Of additional importance, future studies will need to determine if the PvDBP duplication is associated with significant increase (relative to the single-copy PvDBP allele) in levels of PvDBP gene and protein expression. As insufficient materials were available from Duffy-negative infected individuals, testing the association between the PvDBP duplication, or any other candidate alleles, and invasion of Duffy-negative RBC will require development of an adequate study design. Studies of this nature would also provide the opportunity to examine the molecular interactions needed to identify a new erythrocyte receptor for *P. vivax* to engage with in the absence of the Duffy blood group protein.

### Conclusion

We had previously shown that continuing advances in sequencing technology allow robust characterization of single nucleotide polymorphisms in *P. vivax* field isolates. In particular, we showed that high sequence coverage was necessary to identify SNPs and decipher the presence of different strains in a single sample (see Figure 3 from Chan et al [14]). With low coverage (e.g. 20×), it would not have been possible to separate alleles from a strain representing 10% of the *P. vivax* parasites from sequencing errors. Here, we have shown that high coverage sequencing is also important to characterize sequence rearrangements. Since analyses based solely on read coverage are complicated by the complexity of infection observed in patient samples, rigorous identification of sequence rearrangements requires observation of read pairs with unusual organization. Similar to the identification of SNPs, where multiple independent read-pairs need to be observed to exclude stochastic artifactual results, multiple independent read-pairs are also needed to verify the existence of the sequence rearrangements we have identified in this study. Despite a genome coverage >70× in the M15 sample and the fact that the PvDBP was carried by the highly dominant strain in this sample, only 75 read pairs displayed the tail-to-tail orientation indicative of the PvDBP duplication. In the M19 sample the duplication was present on one of the minor strains and only 14 read pairs showed evidence of this diagnostic configuration despite >100× coverage. Obviously, identification of the PvDBP gene duplication in the M19 sample would not have been feasible with lower average sequence coverage (e.g., 20×). Therefore, this observation emphasizes the importance of high sequence coverage for understanding *P. vivax* genomic diversity. The specificity and distribution of the PvDBP duplication may suggest that the parasite genome is responding to the barrier of Duffy negativity through the duplication of this particular gene. Our results raise new questions for understanding alternative pathways for *P. vivax* RBC invasion and the biology of this important malaria parasite.

## Supporting Information

Figure S1 **Schematic representation of possible sequence rearrangements and their resulting read pair organization.** The left panel schematically shows a genomic region in the sample sequenced and one given read pair (small black arrows, representing the sequences from 5′ to 3′). The large green and red arrows indicate the genomic sequences flanking the rearranged DNA sequence (large blue arrow). The right panel shows the resulting mapping on the reference sequence. (**A**) Deletion in the sample sequenced: the read pairs map on the reference genome in the correct orientation (head-to-head) but farther apart than expected based on the reference genome sequence. (**B**) Insertion in the sample sequenced: the arrow originating from the blue sequence in the sample cannot be mapped on the reference genome (since this sequence is missing) resulting in single-end mapping only. (**C**) Inversion in the sample sequenced: the blue sequence is inverted in the sample sequence relative to the orientation in the reference sequence: the paired-ends are mapped on the reference genome in the same orientation (head-to-tail) and farther apart than expected. (**D**) Tandem duplication in the sample sequenced: read pairs overlapping a duplication boundary map in head-to-head orientation in the sample sequenced while they are mapped in the opposite orientation (tail-to-tail) and at the extremes of the duplicated region on the reference genome.
(TIF)

Figure S2 **Read coverage along all the 14 assembled** ***P. vivax*** **chromosomes for three of the samples sequenced.** The innermost red track shows variations in GC content along the genome. The three blacks tracks represent the reads coverage for, from inside to outside, M19, M15 and Belem strains. Note that while the coverage is relatively uniform along most of the chromosome, it shows large variations in telomeric regions.
(TIF)

Figure S3 **Deletion of a Phist gene (PVX_001700) on chromosome 6 in the Belem and C15 samples.** The panels show, for each sample, the number of read pairs per 100 bp (y-axis) that mapped in the correct orientation (head-to-head) and within the expected distance from each other (in blue). The number of read pairs that were separated by more than 1 kb when mapped onto the Sal I reference genome are designated in red. The x-axis corresponds to chromosome 6 between positions 70,100 and 75,100.
(TIF)

Figure S4 **Deletion of the reticulocyte binding protein 2 like gene (PVX_101590) on chromosome 14.** The panels show, for each sample, the number of read pairs per 100 bp (y-axis) that mapped in the correct orientation (head-to-head) and within the expected distance from each other (in blue). The number of read pairs that are separated by more than 1 kb when mapped onto the Sal I reference genome appear in red. The x-axis corresponds to chromosome 14 between positions 3,055,000 and 3,075,000. Note the low level of read coverage at the deleted locus for M19 suggesting that a minor strain in this sample carried the non-deleted allele. See legend to Figure S2 for further details on data analysis and interpretation.
(TIF)

Figure S5 **Duplication of** ***P. vivax*** **Duffy binding protein (PvDBP; PVX_110810) gene on chromosome 6.** The panels show, for each sample, the number of read pairs per 100 bp (y-axis) that mapped head-to-head within the expected distance from each other (in blue) and the number of read pairs that are in tail-to-tail configuration when mapped onto the Sal I reference genome (in red, ×10). Note that, for the M15 sample, the coverage for reads mapping in the expected configuration (blue) roughly doubles and that many reads display a tail-to-tail configuration (red) suggesting that the duplication is carried by the *P. vivax* strain making up most of the parasites in this patient. By contrast, in the M19 sample, the coverage is almost unaffected and very few reads are in tail-to-tail configuration suggesting that only one of the minor *P. vivax* strains carries the PvDBP duplication.
(TIF)

Figure S6 **Reference Allele Frequency in M15 sample.** Each variable nucleotide position observed in a sample is displayed according to the proportion of reads carrying the Sal I reference allele (x-axis). The y-axis shows the number of variable positions with a given RAF.
(TIF)

Figure S7 **Neighbor-joining tree showing the relationships among PvDBP sequences obtained by cloning and Sanger sequencing (full circles) and inferred haplotypes from whole genome sequencing (empty circles) for 5 samples.** Sequences from different samples are represented by different colors. Clustering of M19 and M08 sequences on distinct branches reveals the presence of multiple strains in these samples (with respectively 4 and 2 distinct strains). Note that one of the haplotypes amplified from M19 cluster together with the haplotype sequence from the M15 strain inferred from genome sequence data (black arrow).
(TIF)

Table S1 **Summary of the read mapping using Bowtie 2 for all samples included in this study.**
(XLSX)

Table S2 **All putative rearrangements identified by unusual read pair organization (see Figure S1).**
(XLSX)

Table S3 **Distribution of the PvDBP duplication among Non-travelers (N = 389) and Travelers (N = 110).**
(XLSX)
