# Supplementary figures and images for: Whole Genome Sequencing of Field Isolates Reveals a Common Duplication of the Duffy Binding Protein Gene in Malagasy Plasmodium vivax Strains
Source: PLoS Negl Trop Dis. 2013 Nov 21;7(11):e2489. doi: 10.1371/journal.pntd.0002489 (PMC3836732; doi:10.1371/journal.pntd.0002489)

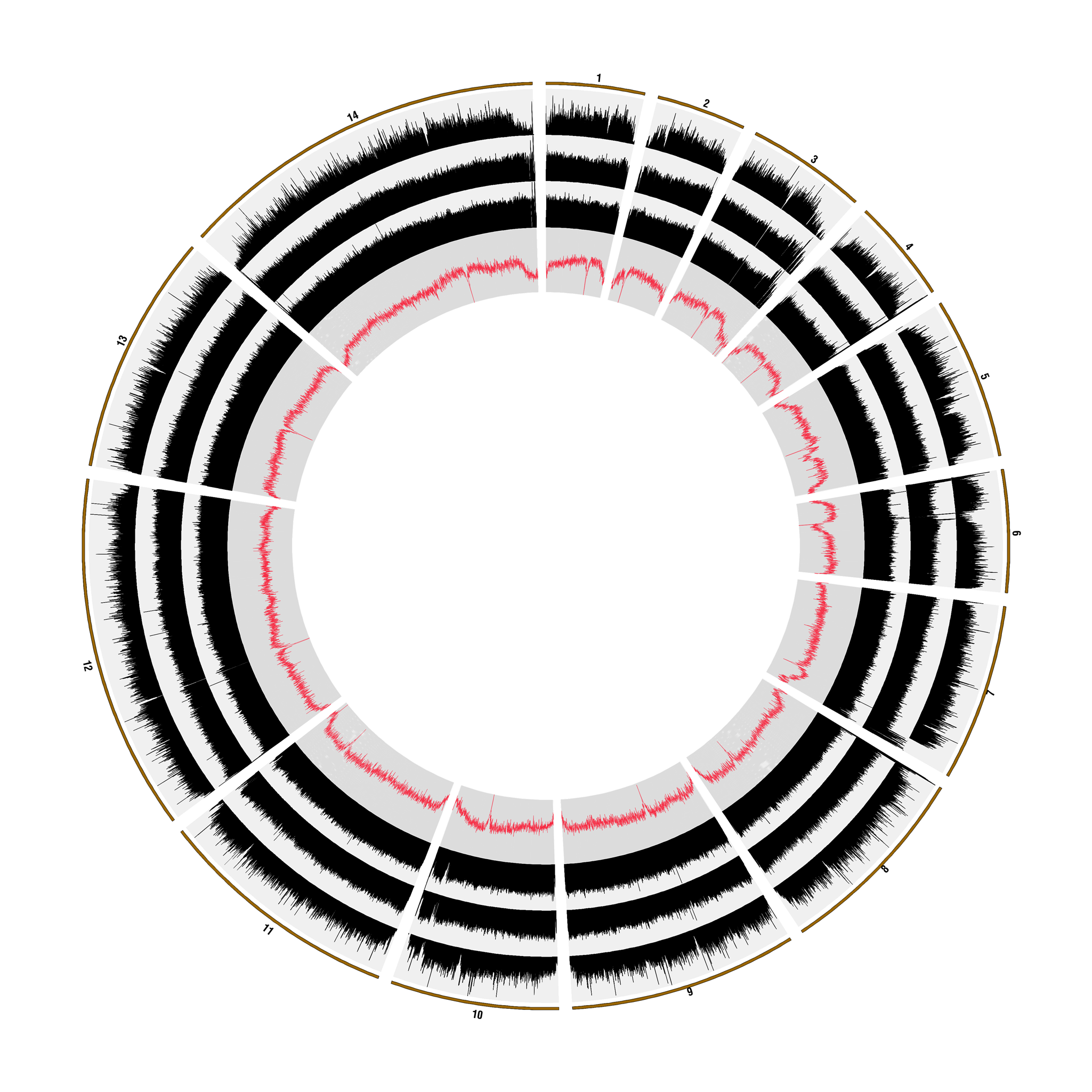

Supplement: Figure S1 — Schematic representation of possible sequence rearrangements and their resulting read pair organization. The left panel schematically shows a genomic region in the sample sequenced and one given read pair (small black arrows, representing the sequences from 5′ to 3′). The large green and red arrows indicate the genomic sequences flanking the rearranged DNA sequence (large blue arrow). The right panel shows the resulting mapping on the reference sequence. (A) Deletion in the sample sequenced: the read pairs map on the reference genome in the correct orientation (head-to-head) but farther apart than expected based on the reference genome sequence. (B) Insertion in the sample sequenced: the arrow originating from the blue sequence in the sample cannot be mapped on the reference genome (since this sequence is missing) resulting in single-end mapping only. (C) Inversion in the sample sequenced: the blue sequence is inverted in the sample sequence relative to the orientation in the reference sequence: the paired-ends are mapped on the reference genome in the same orientation (head-to-tail) and farther apart than expected. (D) Tandem duplication in the sample sequenced: read pairs overlapping a duplication boundary map in head-to-head orientation in the sample sequenced while they are mapped in the opposite orientation (tail-to-tail) and at the extremes of the duplicated region on the reference genome. (TIF) [file pntd.0002489.s001.tif]

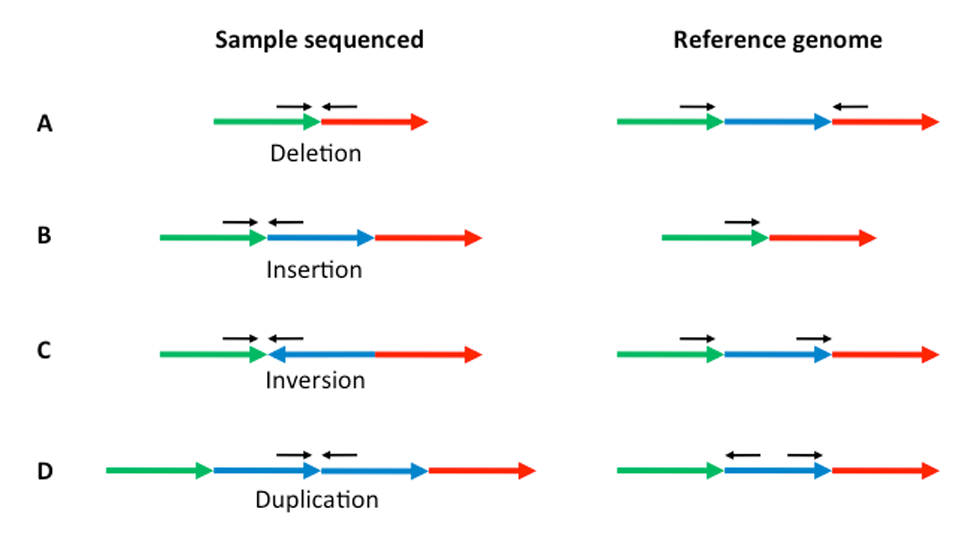

Supplement: Figure S2 — Read coverage along all the 14 assembled P. vivax chromosomes for three of the samples sequenced. The innermost red track shows variations in GC content along the genome. The three blacks tracks represent the reads coverage for, from inside to outside, M19, M15 and Belem strains. Note that while the coverage is relatively uniform along most of the chromosome, it shows large variations in telomeric regions. (TIF) [file pntd.0002489.s002.tif]

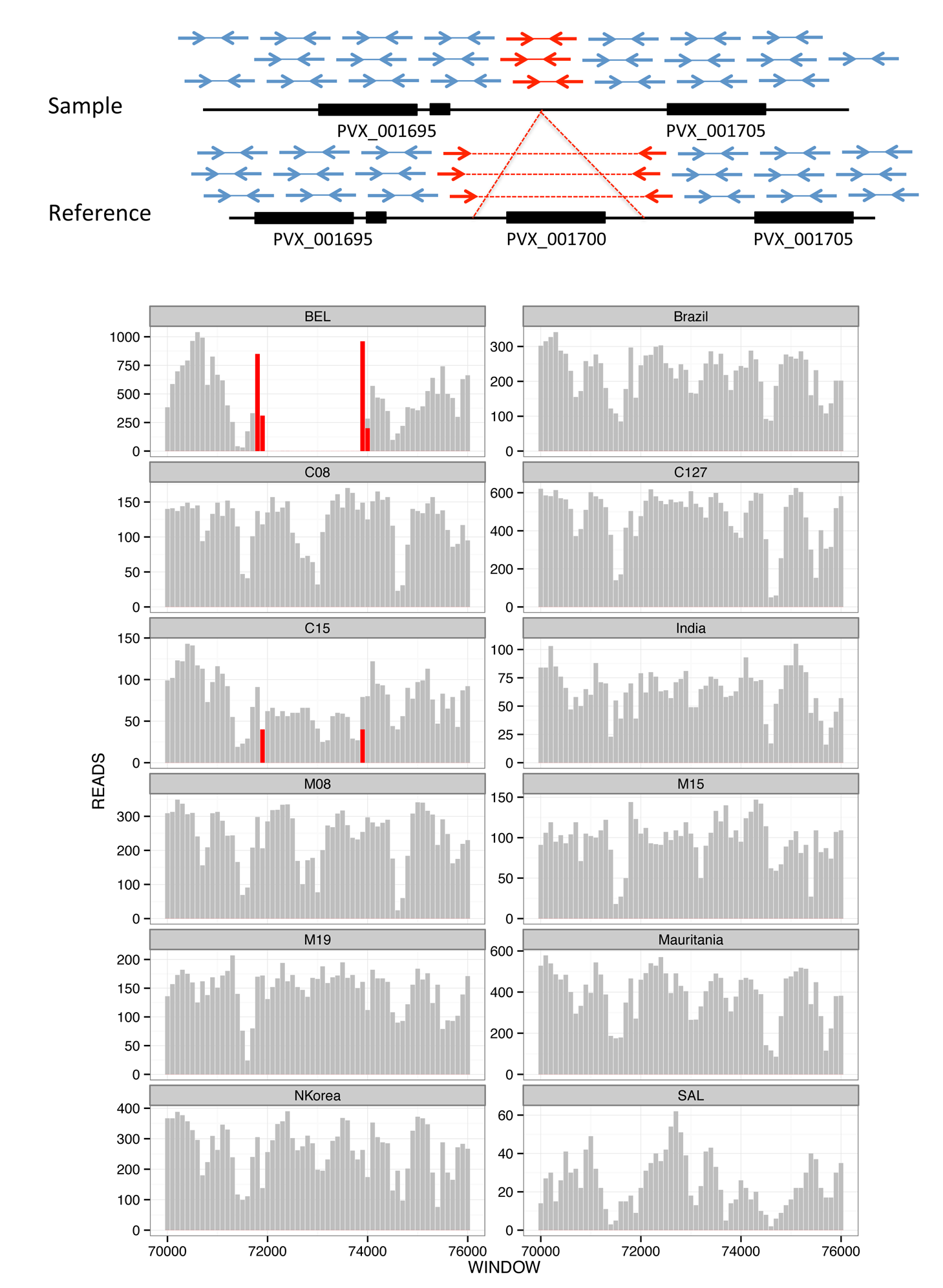

Supplement: Figure S3 — Deletion of a Phist gene (PVX_001700) on chromosome 6 in the Belem and C15 samples. The panels show, for each sample, the number of read pairs per 100 bp (y-axis) that mapped in the correct orientation (head-to-head) and within the expected distance from each other (in blue). The number of read pairs that were separated by more than 1 kb when mapped onto the Sal I reference genome are designated in red. The x-axis corresponds to chromosome 6 between positions 70,100 and 75,100. (TIF) [file pntd.0002489.s003.tif]

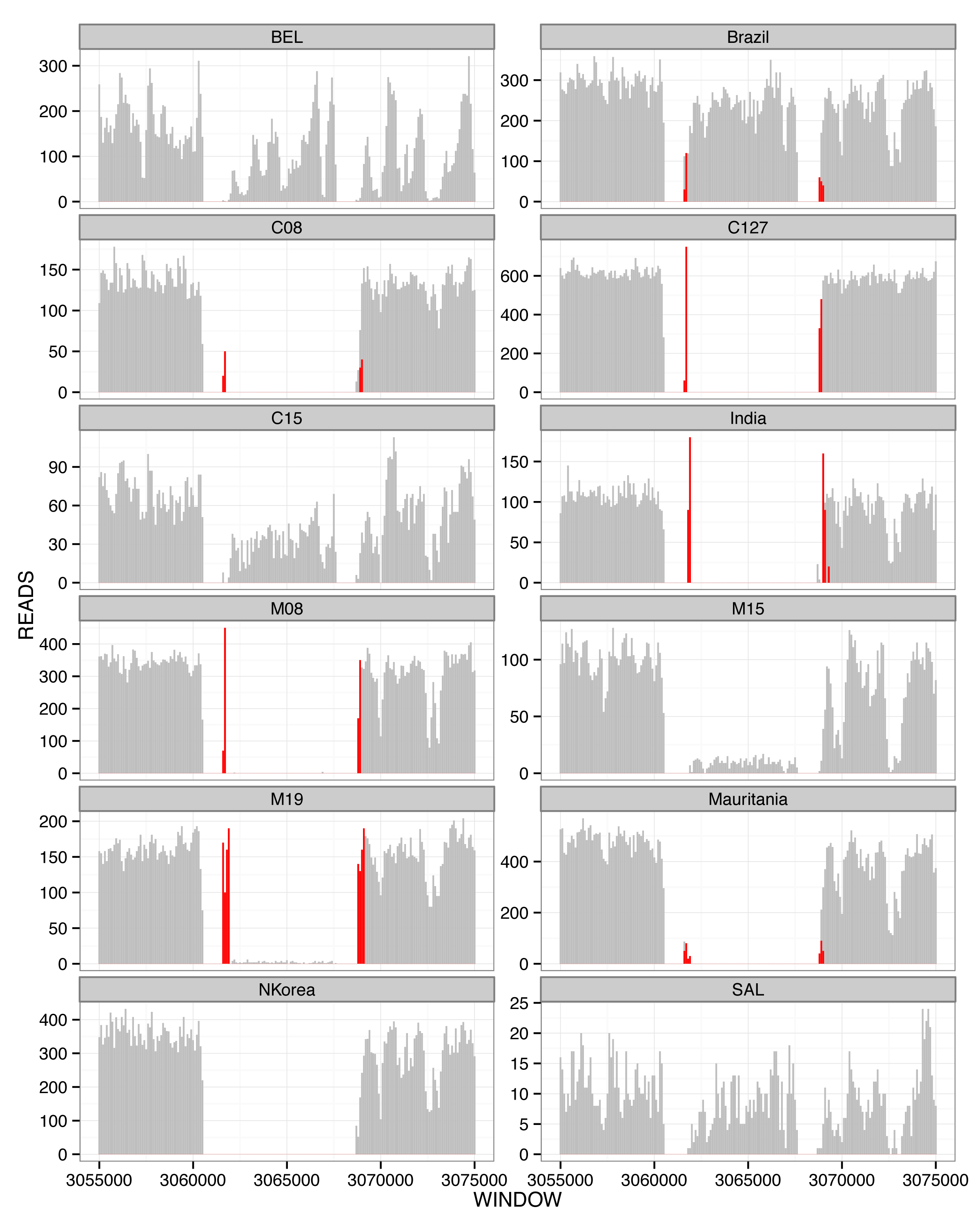

Supplement: Figure S4 — Deletion of the reticulocyte binding protein 2 like gene (PVX_101590) on chromosome 14. The panels show, for each sample, the number of read pairs per 100 bp (y-axis) that mapped in the correct orientation (head-to-head) and within the expected distance from each other (in blue). The number of read pairs that are separated by more than 1 kb when mapped onto the Sal I reference genome appear in red. The x-axis corresponds to chromosome 14 between positions 3,055,000 and 3,075,000. Note the low level of read coverage at the deleted locus for M19 suggesting that a minor strain in this sample carried the non-deleted allele. See legend to Figure S2 for further details on data analysis and interpretation. (TIF) [file pntd.0002489.s004.tif]

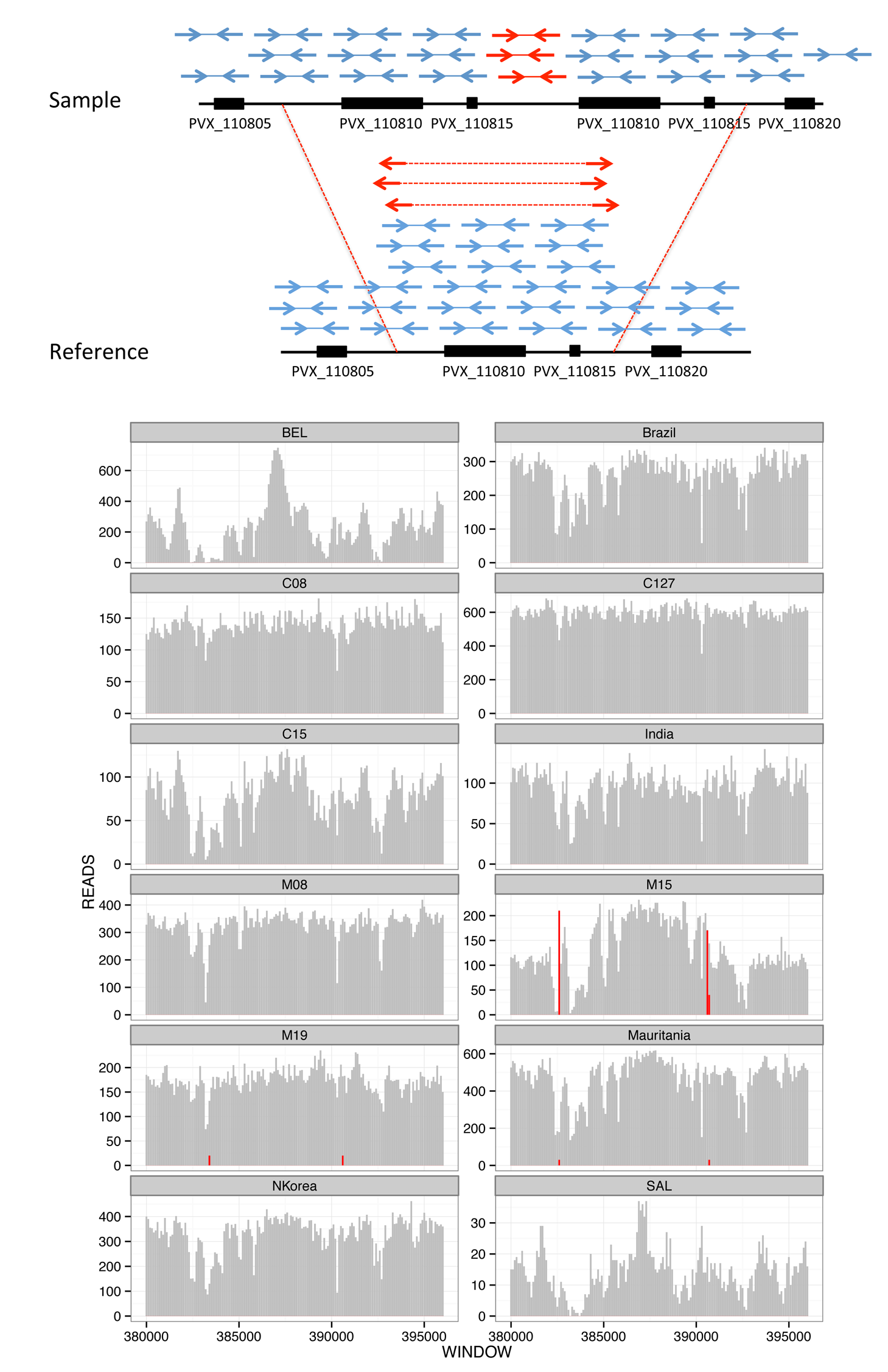

Supplement: Figure S5 — Duplication of P. vivax Duffy binding protein (PvDBP; PVX_110810) gene on chromosome 6. The panels show, for each sample, the number of read pairs per 100 bp (y-axis) that mapped head-to-head within the expected distance from each other (in blue) and the number of read pairs that are in tail-to-tail configuration when mapped onto the Sal I reference genome (in red, ×10). Note that, for the M15 sample, the coverage for reads mapping in the expected configuration (blue) roughly doubles and that many reads display a tail-to-tail configuration (red) suggesting that the duplication is carried by the P. vivax strain making up most of the parasites in this patient. By contrast, in the M19 sample, the coverage is almost unaffected and very few reads are in tail-to-tail configuration suggesting that only one of the minor P. vivax strains carries the PvDBP duplication. (TIF) [file pntd.0002489.s005.tif]

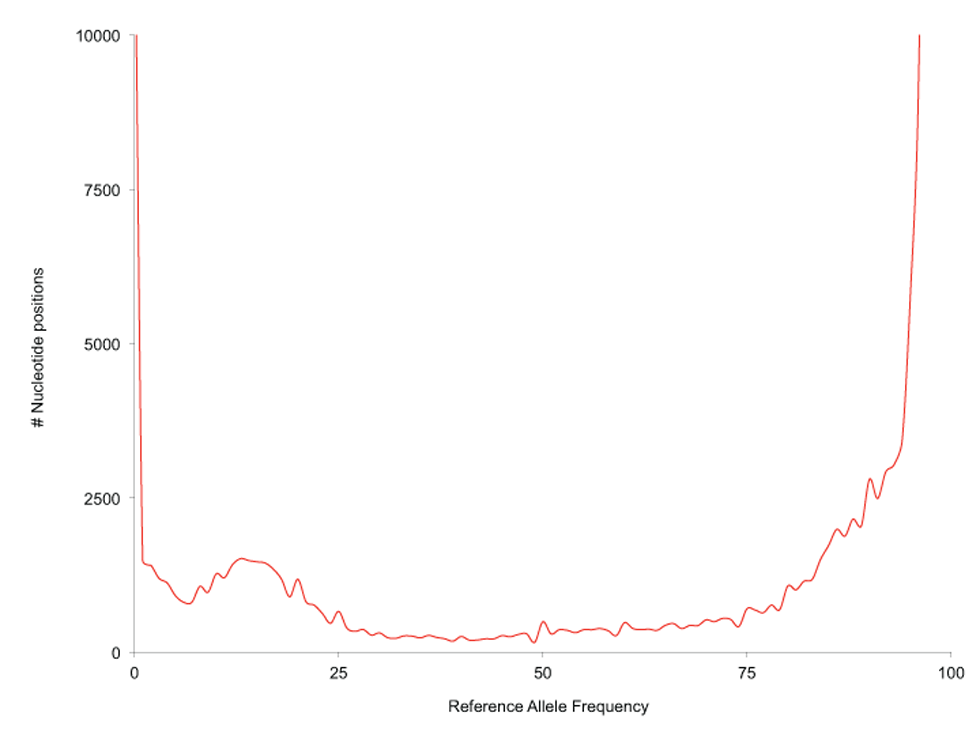

Supplement: Figure S6 — Reference Allele Frequency in M15 sample. Each variable nucleotide position observed in a sample is displayed according to the proportion of reads carrying the Sal I reference allele (x-axis). The y-axis shows the number of variable positions with a given RAF. (TIF) [file pntd.0002489.s006.tif]

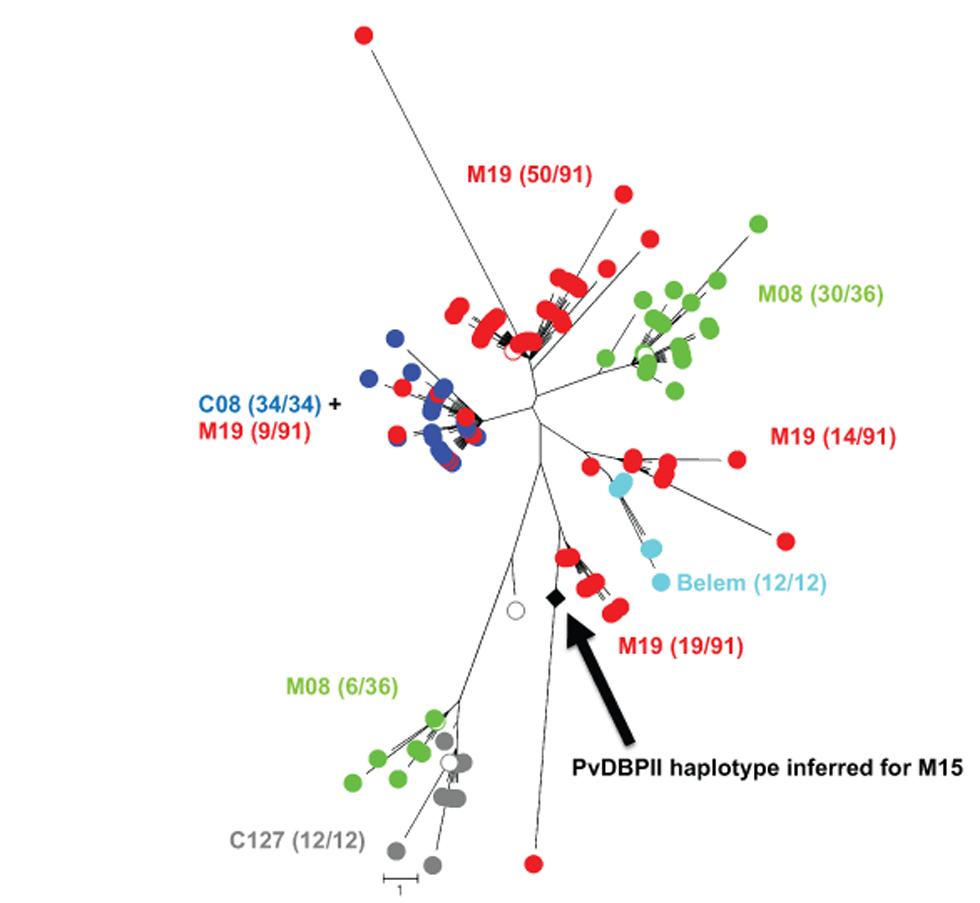

Supplement: Figure S7 — Neighbor-joining tree showing the relationships among PvDBP sequences obtained by cloning and Sanger sequencing (full circles) and inferred haplotypes from whole genome sequencing (empty circles) for 5 samples. Sequences from different samples are represented by different colors. Clustering of M19 and M08 sequences on distinct branches reveals the presence of multiple strains in these samples (with respectively 4 and 2 distinct strains). Note that one of the haplotypes amplified from M19 cluster together with the haplotype sequence from the M15 strain inferred from genome sequence data (black arrow). (TIF) [file pntd.0002489.s007.tif]
